# Feasibility of Autofluorescence Using Overlay Imaging for the Detection of Parathyroid Glands: Defining Standards

**DOI:** 10.1245/s10434-023-14552-7

**Published:** 2023-11-13

**Authors:** Melisa Arikan, Josef Hegazy, Sophie Mertlitsch, Teresa Binter, Lindsay Hargitai, Christian Scheuba, Philipp Riss

**Affiliations:** Division of Visceral Surgery, Department of General Surgery, Medical University of Vienna, Vienna, Austria

**Keywords:** Autofluorescence, Parathyroid glands, Overlay imaging, Near-infrared light, Near-infrared (auto)fluorescence

## Abstract

**Background:**

The aim of this study is to define standards for the use of near-infrared autofluorescence (NIRAF)-based overlay imaging via EleVision IR (Medtronic, Dublin, Ireland) and to evaluate its clinical applicability.

**Patients and Methods:**

This prospective study included 189 patients who had undergone open thyroid and/or parathyroid surgery and in whom EleVision IR was applied to visualize at least one parathyroid gland (PG) between January 2021 and May 2022 in a tertiary referral care center. Whether the PGs were first localized by the surgeon or by overlay imaging was noted. Handling of the device, application time and duration, distance, infrared intensity (IR%), and the angle of each measurement were analyzed. In thyroidectomies, the specimens were subsequently scanned for further PGs. NIRAF patterns and intensities were described.

**Results:**

Overall, 543 PGs were analyzed in 158 (83.6%) surgeries of thyroid glands (TGs) and in 49 (25.9%) surgeries for hyperparathyroidism. In 111 (58.7%) patients, identical numbers of PGs were detected by the surgeon and by overlay imaging. While a larger number of PGs was identified by the surgeon in 48 (25.4%) patients, overlay imaging served to detect more PGs in 30 (15.9%) cases. In four (2.1%) patients, PGs were visualized post-thyroidectomy due to their autofluorescence on the specimen. NIRAF-based overlay imaging was applied to depict the PGs early on after exposure by the surgeon. The ideal distance for the measurement ranged between 8 and 12 cm with an angle of 90° and a mean IR% of 34.5% (± 17.6).

**Conclusions:**

Considering the standard operating procedures, NIRAF-based overlay imaging can be used as an adjunct tool for intraoperative localization.

Postoperative hypoparathyroidism, which occurs temporarily in up to 33% and permanently in up to 8% of cases, still represents a major problem in thyroid surgery.^1–5^ In order to reduce this complication, near-infrared autofluorescence (NIRAF) is increasingly used to intraoperatively localize parathyroid glands (PGs).^6–9^

EleVision IR (Medtronic, Dublin, Ireland) represents a development of NIRAF imaging, which differs from current products in its (threshold) overlay imaging resulting from white and near-infrared (NIR) light overlapping. The use of EleVision IR was first demonstrated by Kamada et al. in a case report on indocyanine green (ICG)-guided parathyroidectomy.^8^ Preliminary experience was described by Makovac et al.^10^

Currently, there are no standards for the use of NIRAF-based overlay imaging for intraoperative PG detection. Thus, our prospective study was conducted to define standards for the usage of EleVision IR in endocrine neck surgery and to demonstrate its feasibility in clinical practice.

## Patients and Methods

All patients having undergone open thyroid and/or parathyroid surgery and in whom NIRAF imaging via EleVision IR was used to visualize at least one PG between January 2021 and May 2022 at the Medical University Hospital of Vienna, Department of General Surgery, Division of Visceral Surgery, were included in this prospective study in accordance with the Austrian Medical Device Act.

All parameters were documented consecutively and pseudonymized in a database. Initial cases, in which NIRAF-based overlay imaging was intraoperatively applied, were included in this study.

### NIRAF-Based Overlay Imaging

**Fig. 1:**
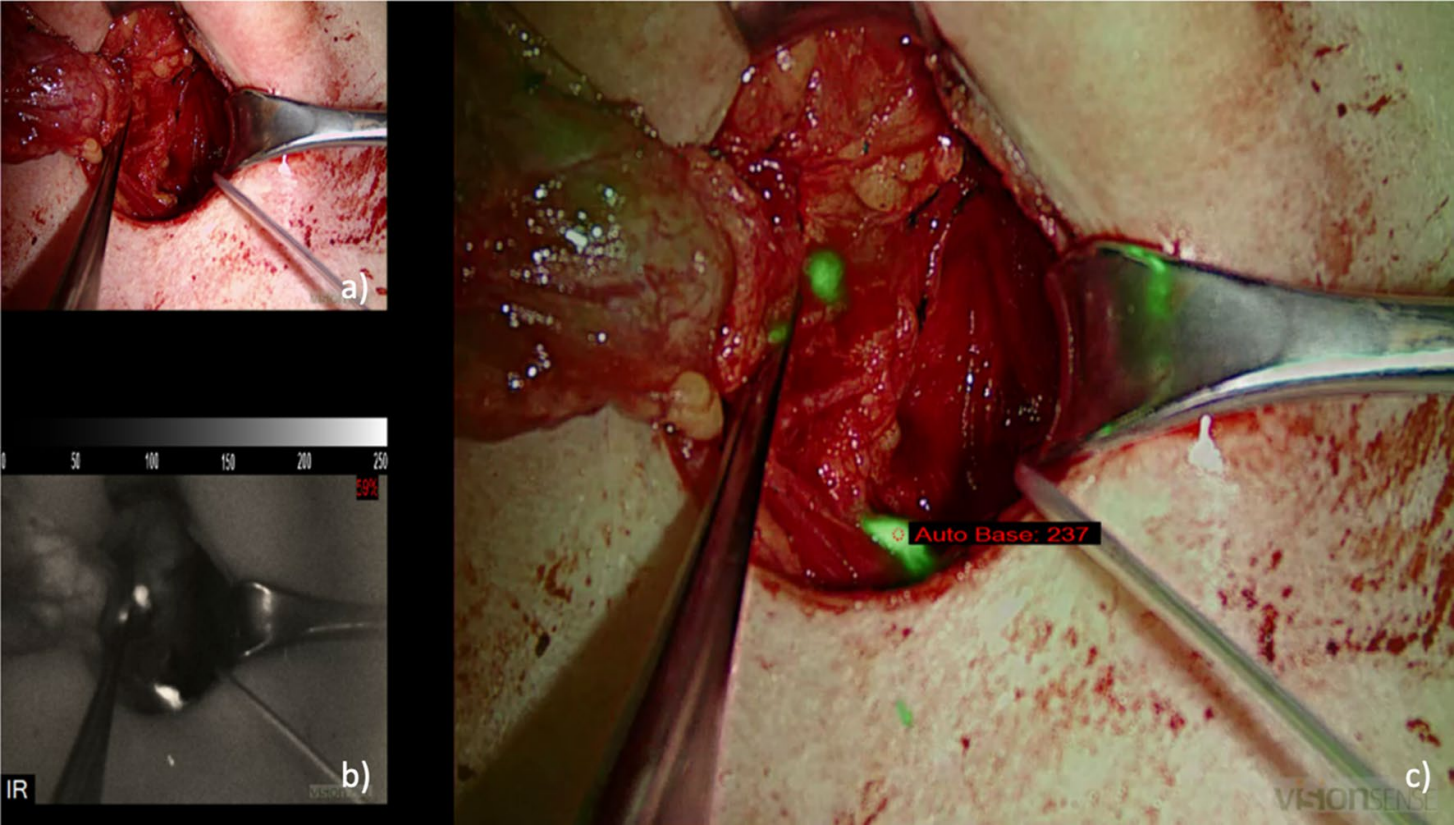
**a** White light imaging, **b** NIRAF imaging, **c** NIRAF-based overlay imaging

EleVision IR was applied in our study to measure NIRAF. After activation of the diode laser, light with a wavelength of 785 nm was emitted onto the PG and resulted in excitation of endogenous fluorophores. Light signals were then sent back to a miniature microscope covering an emission spectrum of 825 to 850 nm. Thus, three image views were obtained: white light, NIR, and overlay images, as shown in Fig. 1. The last mentioned consists of an overlapping of white and NIR light and leads to a green display of fluorescent tissue. Further, EleVision IR features an automatic adjustment mechanism for the intensity of excitation light (IR%) and an NIR overlay threshold function.

### Intraoperative Application

At the beginning of the surgery, the NIRAF-based overlay imaging device was placed on the right side of the patient’s body next to the surgeon. For surgeons operating from the left side of the patient, the device was nevertheless left on the patient’s right side. Before EleVision IR was used, the diode laser including its arm was covered with a sterile drape, since the camera had to be placed above the operating field. One of two highly experienced surgeons initially identified the PGs. Subsequently NIRAF-based overlay imaging was used.

If necessary, the image of the NIRAF-based overlay device could be flipped easily by 180° by pressing a button on the screen of the device or on its sterile arm.

The distance between the PGs and the device was established with a measuring tape during each measurement. Whether PGs were first visually localized by the surgeon or by overlay imaging was noted intraoperatively. Following thyroidectomy, the specimen was subsequently scanned for further PGs. In case of difficult PG localization, the threshold adjustment function of EleVision IR was applied.

While the pathologically altered PGs were analyzed in final histology, the healthy PGs were not sent to histology due to ethical reasons. Structures other than PGs, which could not be identified clearly during surgery but showed an autofluorescence pattern, were sent to frozen section.

During each measurement, the operating room (OR) lights and surgical headlights, if in use, were left on. In turn, the operating lights [light-emitting diodes (LED)] were turned off. The measurements were saved automatically and can be displayed at any time.

### Analyzed Parameters

The demographic data include age and sex (male, female). Intraoperative data, such as surgery duration (in minutes) and performed surgery [thyroidectomy, hemithyroidectomy, subtotal thyroidectomy, open minimally invasive parathyroidectomy (OMIP), unilateral and bilateral exploration, and subtotal and total parathyroidectomy], were documented. Further, autotransplantation of PGs (APTX) was documented. Data associated with NIRAF-based overlay imaging, such as duration of handling, mean distance, IR%, the angle during the measurement, and number of identified PGs by the surgeon and by overlay imaging were analyzed. Interrater reliability between the surgeon and EleVision IR in detecting PGs was calculated.

The postoperative data included the final histological results of thyroid [nodular goiter, Grave’s disease, thyroiditis, papillary, follicular, medullary and Hürthle cell carcinoma, noninvasive follicular thyroid neoplasm with papillary-like nuclear features (NIFTP), follicular tumor of uncertain malignant potential (FT-UMP)] and parathyroid specimens (parathyroid adenoma, parathyroid hyperplasia). The postoperative mean values of calcium and parathyroid hormone (PTH) were described.

### Statistical Analyses

Data analysis was performed using SPSS^®^version 23.0 (SPSS^®^,Chicago,Illinois). Data at a level of *p* < 0.05 were considered statistically significant. The number of patients required to identify a statistically significant difference/value was calculated using G*Power (Heinrich Heine University, Düsseldorf, Germany).

Metric parameters (such as distance, angle, and IR%) were described by mean values, the corresponding standard deviation, minimum, and maximum. To define the ideal distance, a range of the most frequently used distance values was determined.

Ordinal and nominal parameters were described by absolute and relative numbers. Interrater reliability was calculated using Cohen’s kappa.

Standards for device placement, sterile draping and application time of the NIRAF-based overlay device were developed based on experience considering intraoperative efficiency and patient safety.

### Ethics Approval

This study was approved by the Ethics Committee of the Medical University of Vienna (EK 1345/2021). All patients gave their written informed consent for all diagnostic and therapeutic procedures.

## Results

### Demographic Data and Surgeries

In total, 189 patients, 141 (74.6%) female and 48 (25.4%) male, mean age 50.9 ± 16.9 (5–85) years, were included in this study, as presented in Table 1. Mean surgery duration was 85 ± 39.5 (10–290) minutes.

**Table 1:** Demographic and intraoperative data of patients (*n* = 189)

	Mean ± SD (range)		*n* (%)
Age	50.9 ± 16.9 (5–85)	Sex	
		Female	141 (74.6%)
		Male	48 (25.4%)
Surgery time	85 ± 39.5 (10–290)		

	*n* (%)
Analyzed nerves at risk	319
Analyzed PGs	543
Surgery of TG	
Thyroidectomy	120 (63.5)
With neck dissection	15 (7.9)
Hemithyroidectomy	34 (18)
Subtotal thyroidectomy	3 (1.6)
Surgery of PG	
OMIP	31 (16.4)
Unilateral exploration	24 (12.7)
Bilateral exploration	13 (6.9)
Subtotal parathyroidectomy	2 (1.1)
Total parathyroidectomy	2 (1.1)
Combined surgery TG and PG	18 (9.5)
Autotransplantation of PG	
Due to thyroidectomy	11 (5.8)
With neck dissection	2 (1.1)
Due to tHPT	2 (1.1)

*SD* standard deviation, *PG* parathyroid gland, *TG* thyroid gland, *OMIP* open minimally invasive parathyroidectomy, *tHPT* tertiary hyperparathyroidism

Overall, 543 PGs were analyzed in 120 (63.5%) thyroidectomies, including 15 (7.9%) neck dissections, 34 (18%) hemithyroidectomies, 3 (1.6%) subtotal thyroidectomies, and 49 (25.9%) PG surgeries. Of the patients who underwent parathyroid surgery, OMIP, unilateral exploration and bilateral exploration were performed in 24 (12.7%), 8 (4.2%) and 13 (6.9%) patients, respectively, due to primary hyperparathyroidism (pHPT). In two (1.1%) patients each, subtotal and total parathyroidectomy with APTX was performed due to secondary and tertiary hyperparathyroidism (sHPT and tHPT), respectively. Further, in 18 (9.5%) patients, surgery of TGs and PGs was combined.

**Fig. 2:**
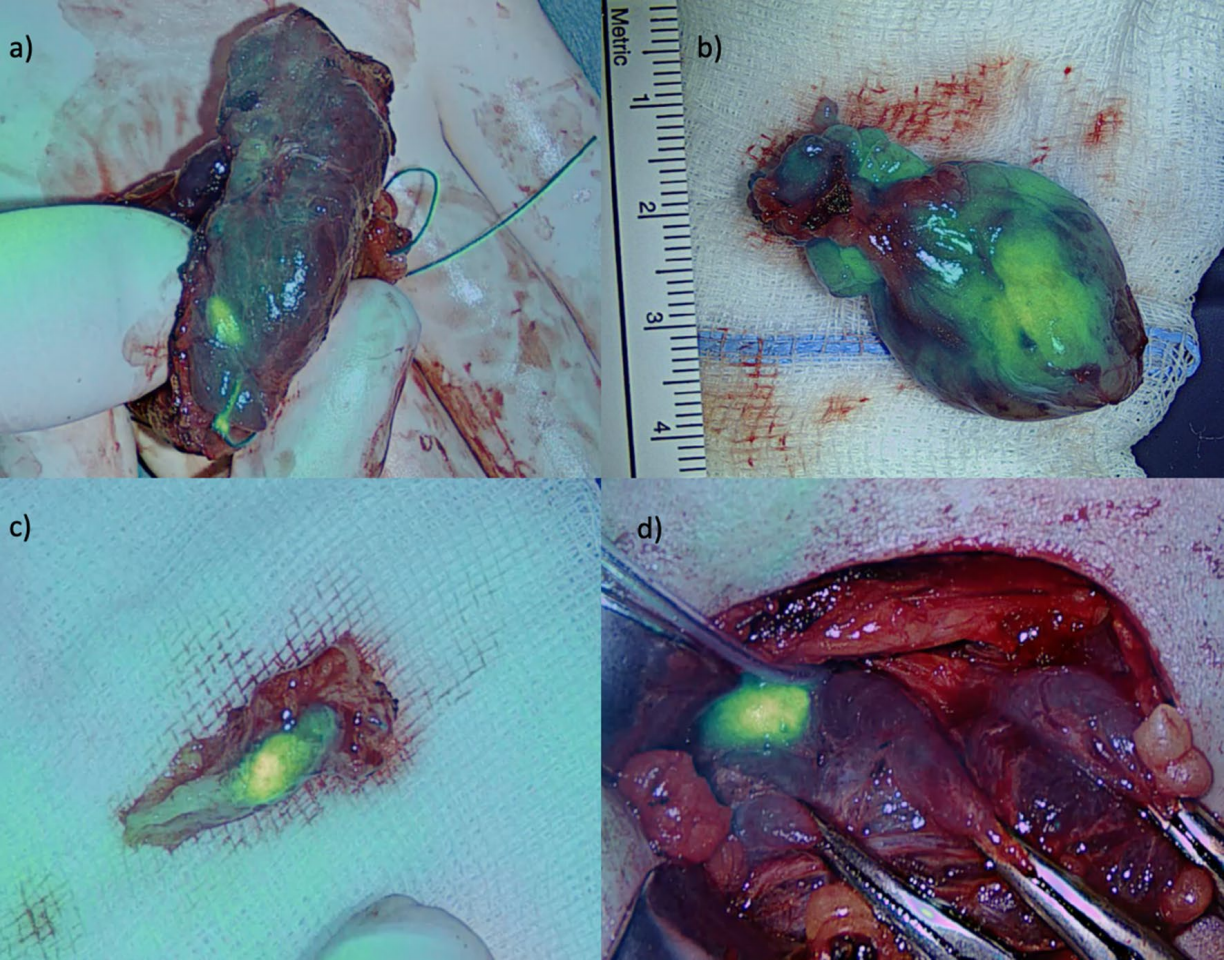
Various autofluorescence patterns of PGs. **a** PG on thyroid gland after thyroidectomy in NIRAF-based overlay imaging. **b** Adenoma of the PG ex vivo in NIRAF-based overlay imaging. **c** Cut surface of PG adenoma ex-vivo. **d** PG hyperplasia in tHPT in NIRAF-based overlay imaging

APTX was necessary in 15 (7.9%) patients. Intraoperative APTX was necessary in 13 (6.9%) thyroidectomies involving two (1.1%) neck dissections. In four (2.1%) of these patients, PGs were visualized on the specimens by using EleVision IR as depicted in Fig. 2a. Two patients received APTX due to total parathyroidectomy in tHPT, as depicted in Table 1.

The mean postoperative PTH value was 25.24 ± 21.9 (1.2–159) pg/mL and the mean postoperative calcium level was 2.19 ± 0.15 (1.7–2.6) mmol/L.

### Histology

Benign TG histology was present in 122 (59%) patients, including 78 (37.5%) nodular goiters, 14 (6.8%) adenoma of the TG, 22 (10.6%) cases of Graves’ disease, 8 (3.9%) cases of thyroiditis, one (0.5%) patient with NIFTP, and two (1%) with FT-UMP. In 33 (15.9%) patients, differentiated thyroid carcinoma including 5 (15.2%) papillary thyroid microcarcinoma, 17 (51.5%) papillary thyroid, 2 (6.1%) follicular, 4 (12.1%) medullary and 5 (15.2%) Hürthle cell thyroid carcinoma, respectively, were identified, as presented in Table 2.

**Table 2:** Histology

	*n* (%)
Benign	
Nodular goiter	78 (37.5)
Adenoma of the thyroid gland	14 (6.8)
Grave’s disease	22 (10.7)
Thyroiditis	8 (3.9)
NIFTP	1 (0.5)
FT-UMP	2 (1)
DTC	
PTC	17 (16.1)
mPTC	5 (15.2)
MTC	4 (12.1)
FTC	2 (6.1)
Hürthle cell TC	5 (15.2)
PGs	
Adenoma	32 (15.5)
(Multigland) hyperplasia	17 (8.2)

*NIFTP* noninvasive follicular thyroid neoplasm with papillary-like nuclear features, *FT-UMP* follicular tumor of uncertain malignant potential, *DTC* differentiated thyroid carcinoma, *PTC* papillary thyroid carcinoma, *mPTC* papillary thyroid microcarcinoma, *MTC* medullary thyroid carcinoma, *FTC* follicular thyroid carcinoma

Histological PG analysis revealed adenoma in 32 (15.5%) cases and (multigland) hyperplasia in 17 (8.2%) patients.

### Defining Standards

#### Surgeon versus EleVision IR

The mean number of PGs visualized in this study per patient, either by the surgeon or by NIRAF imaging, was 2.8 ± 1.2 (0–4). The mean number of PGs detected by the surgeon was 2.3 ± 1.3 (0–4) and that of PGs visualized by EleVision IR was 2.1 ± 1.3 (0–4). In 111 (58.7%) patients, the surgeon and application of autofluorescence imaging detected the same numbers of PGs in each patient. In 48 (25.4%) patients, the surgeon detected more PGs than was achieved with overlay imaging. In 30 (15.9%) cases, using NIRAF-based overlay imaging served to identify more PGs, as depicted in Table 3.

**Table 3:** Surgeon-versus NIRAF-based imaging: identified PGs per patient

	Mean ± SD, range		*n* (%)
Surgeon	2.3 ± 1.3 (0–4)	Surgeon > EleVision IR	48 (25.4)
EleVision IR	2.1 ± 1.3 (0–4)	Surgeon = EleVision IR	111 (58.7)
		Surgeon < EleVision IR	30 (15.9)

*SD* standard deviation

The interrater reliability of detecting PGs between the surgeon and overlay imaging showed a moderate accordance with a Cohen’s kappa value of 0.46 and a statistically significant result of *p* < 0.001.

#### Standards

One of two experienced endocrine surgeons initially identified the PGs after TG mobilization using magnifying loupes. Subsequently, NIRAF-based overlay imaging was used to either confirm visual PG identification by the surgeon before performing any substantial attempts to identify PGs or to search PGs if that initial identification by the surgeon proved unsuccessful. Further dissection of the surrounding tissue was performed before reapplication of EleVision IR. During each measurement, the IR% was left on automatic. Thus, depending on the surrounding tissue, the strength of the excitation light was adjusted automatically.

Divergence between the measured distance and the distance appearing on the display was noticed. The mean distance was 9.6 ± 29.5 (5–20) cm. The ideal distance for the measurement ranged between 8 and 12 cm with a mean IR% of 34.5 ± 17.6 (2–119), as depicted in Table 4. In case of difficult PG localization, we made use of the threshold adjustment function of EleVision IR but failed to experience an advantage in localizing PGs.

**Table 4:** Recommendations for optimal use

1. Positioning of the device on the right side of the patient (even for surgeons operating from the left side of the patient). Application feasible either by assistant or surgeon	
2. Covering the diode laser and the arm of the device with a sterile drape	
3. After mobilization of the thyroid gland, identification of the PG by the surgeon.	
4. Turning off surgery lights due to LED interference with the diode laser	
5. Confirmation of localized PG by using EleVision IR	
6. Measurement of NIRAF of the target structure with a distance of 8 to 12 cm and an angle of 90° between diode laser and target tissue. If necessary, image can be flipped by 180°	
7. If identification of target structure is unsuccessful, slight dissection of the surrounding tissue is recommended before re-application of EleVision IR	
8. Following thyroidectomy, scanning of the specimen for further PGs is recommended	

#### Autofluorescence Pattern and Intensity

Intact PGs showed a homogeneous distribution of NIRAF throughout the tissue. In turn, in PG adenoma, we observed a heterogeneous NIRAF pattern with a higher level of intensity in non-adenomatously modified tissue and lower intensity in adenomatous tissue, as seen in Fig. 2b, c. In patients with (multigland) PG hyperplasia due to pHPT, stronger illumination was present than in those with PG adenoma, as depicted in Fig. 2d. Further, hyperplasia of PGs due to renal hyperparathyroidism (rHPT) presented very low NIRAF intensity.

In brown adipose tissue, we observed a very similar and strong NIRAF pattern as in nonpathologically altered PGs. NIRAF intensity of the TG was stronger in thyroiditis than in other pathologically altered TGs but was still less than seen in healthy PGs.

Metastasis of medullary thyroid carcinoma (MTC) and thyroid cysts (either hemorrhaged or with detritus) showed a similar NIRAF pattern and intensity as did intact PGs. Further observation confirmed autofluorescence with strong intensity of Ethicon VICRYL suture material as seen in Fig. 2a.

### Learning Curve

In the first 10–20 patients, the application duration of NIRAF-based overlay-imaging was 5 minutes per PG. In those patients, we experienced a wide range of various NIRAF patterns on different tissues. The distance of each measurement and IR% varied likewise. In our first cases we performed measurements using NIRAF-based overlay imaging with and without OR lights turned off. We observed no significant difference in PG depiction during the measurement with the OR lights turned off.

After the 20th patient, the imaging of single PGs took up to 30 seconds. From these patients on OR lights were left on. After 50 to 100 patients, 5 to 10 seconds were necessary for the intraoperative measurement of NIRAF per PG. Routinized application was achieved after 70 patients.

## Discussion

Over the past years, NIR fluorescence (NIRF) and NIRAF imaging have been further developed in an attempt to reduce the risk of postoperative hypoparathyroidism after endocrine neck surgery.^6–8,11–18^

Paras et al. were the first to describe NIRAF in 2011 as a nonintrusive, real-time intraoperative imaging tool for PG detection. In all patients (*n* = 21) included in their analysis, parathyroid tissue exhibited more intense autofluorescence after exciting the tissue with NIR light (785 nm wavelength) with a diode laser. Thus, the PGs could be distinguished intraoperatively from surrounding tissue.^13^ This technology is still used for NIRAF imaging of parathyroid tissue, with a large number of different probe- and image-based devices.^7^

Sarder et al. were the first to demonstrate overlay imaging in oncologic surgery in 2013,^19^ which was subsequently investigated in animal models^20,21^ before the usage of the Overlay Tissue Imaging System (OTIS) was published by McWade et al. in 2019 for the detection of PGs.^16,18^ OTIS serves to collect tissue NIRAF signals and projects them as a visible overlay image onto the operative field but without any remote display monitor.^16^

EleVision IR is a development of NIRAF-based overlay imaging generating an intraoperative real-time image on a display monitor. In 2020, Kamada et al. introduced the application of overlay imaging via EleVision IR in a case report of ICG-guided parathyroidectomy. In the mentioned study, advantage was taken of the overlay threshold function to localize parathyroid adenoma.^8^ In 2022, preliminary experience with 25 patients was published by Makovac et al.^10^

In our prospective study, we focused our attention on setting standards for the usage of NIRAF-based overlay imaging via EleVision IR to detect PGs (both healthy and affected) and to demonstrate its clinical applicability. Our data analysis is the first study to define standards for the usage of NIRAF-based overlay imaging using EleVision IR.

### Demographic Data and Surgery Duration

More than 50% of the patients included in our analysis were female, as presented in Table 1. Thus, sex-specific differences could not be considered due to gender bias while defining standards for overlay imaging, which is similar to previous studies using NIRAF for PG localization.^11,22–24^

In our patient collective, we detected a slight change in surgery time, mainly in the first 20 cases, due to the application duration of the NIRAF device. Rapid and routinized application seems to be achieved after 70 patients with an application duration of 5 to 10 seconds and correct identification of NIRAF patterns. Mean surgery time was 85 ± 39.5 (10–290) minutes. In a randomized clinical trial, Benmiloud et al. described significantly longer surgery durations in the NIRAF group using the Fluobeam 800 system (Fluoptics, Grenoble, France) than in the control group, in which PGs were localized with the naked eye.^25^ Differences in device handling could explain this result.

### Standards

Our results showed a moderate degree of accordance for the detection of PGs between the surgeon and overlay imaging. Thus, NIRAF-based overlay imaging has a potential to detect PGs similar to that of experienced surgeons and can be used as an adjunct tool. However, despite the similar results of PG localization, there are still differences in the ability to identify PGs. In 25.4% of the patients included in the study, more PGs were detected by the surgeon than with overlay imaging. The surgeon was fully confident in identifying all those PGs. In 15.9% of the patients, overlay imaging served to detect more PGs and visualized PGs on the specimen in four (2.1%) thyroidectomized patients. Similar results were reported.^10^

During each measurement, the OR and surgical headlights, if in use, were left on. In turn, the surgery lights were turned off due to LED interference with the EleVision IR diode laser. No significant difference was observed in PG depiction during the measurement with the OR and surgical headlights turned off. In contrast to our observations, McWade et al. described that during each measurement using OTIS, all OR and surgical headlights were switched off to minimize light interference.^16^ For other devices described in the current literature, OR lights were likewise turned off.^7,11,16,23^ In contrast to our results, Makovac et al. turned off or covered all light sources while using EleVision IR.^10^

During each measurement, we placed the NIRAF-based overlay imaging device on the right side of the patient opposite the instrumentalist table. The application of the camera was applied by either the surgeon or the assistant. In another study using EleVision IR, the device was positioned above the patient or left on the surgical instrument table.^10^ This difference in placement can be explained by the fact that the surgeon who introduced EleVision IR in our department is left-handed and operates from the left side of the patient. For surgeons operating from the right side of the patient, the camera is placed by the assistant.

The display with real-time images can be positioned at a required angle by the surgeon due to the mobility of the display. Highly informative real-time imaging on the display makes it easier to interpret the images, even for endocrine surgeons in training. In contrast, different types of NIRAF-based images exist in the current literature, such as projection of the NIRAF image onto the operative field, black-and-white image, and information about the measured tissue on the display but without any image. Using those devices, results can be interpreted incorrectly without adequate operative experience.^15–18,26,27^

We recognized a divergence between the measured distance and the distance appearing on the display of the NIRAF device. During each measurement, the distance was automatically calculated between the laser and the closest tissue to the laser, which in many cases was the skin. Thus, the distance was measured manually. Our results yielded a mean “optimum” distance of 9.6 cm between the localized tissue and the laser. In our experience, the best distance for PG localization was 8 to 12 cm with an ideal angle between the camera and tissue of 90°. The range covered 5 to 20 cm, whereas our initial results were included in these calculations. Depending on the device applied, distances of up to 35 cm using NIRAF-based imaging have been described in the current literature.^11,16,18^

Despite keeping the ideal distance and angle for the detection of PGs, overlay imaging was more difficult if they were covered with adipose tissue. In those cases, the automatic adjustment of IR% showed an advantage in intraoperatively localizing PGs. Mean IR% showed a value of 34.5 ± 17.6%. The more the PGs were covered by adipose tissue, the greater IR% values were present. Further, slight dissection of the PG was performed in the event that IR% adjustment for detection was insufficient. Preliminary experience published by Makovac et al. showed a distance of 20 cm to the targeted tissue. PG localization was first performed with the naked eye using magnifying loupes. EleVision IR was used either to confirm PGs or to find them if initial localization was unsuccessful. The grade of dissection of the surrounding tissue was not described in detail.^10^ In comparison to our study, Kamada et al. mainly made use of the overlay threshold adjustment function to discard NIR signal values below 50%, resulting in clear PG contours in ICG-guided parathyroidectomy.^8^

During some measurements, when the PGs were difficult to localize, we made use of the threshold adjustment function but failed to experience an advantage in NIRAF-based overlay imaging. Beside these insights, no further statement on the ideal usage of overlay imaging via EleVision IR was made.

Considerable advantage was offered by automatically saving all measurements, which at any time can be recalled and used for research. Further, various measurement points of different intensity can be measured again postoperatively.

### Autofluorescence Intensity and Patterns

The results of our study showed autofluorescence intensity and patterns to be distributed differently in various thyroid and parathyroid diseases in the course of applying NIRAF-based overlay imaging via EleVision IR to localize PGs. These observations had been published before using other NIRAF image devices.^11,12,15,28,29^

In this study, the best NIRAF intensity and most homogenous pattern were present in healthy PGs. Similar results have been described in the current literature using other NIRAF-based devices.^11,13,17^

Furthermore, brown adipose tissue presented a similar NIRAF intensity and pattern as did healthy PGs, which is comparable to findings described by DeLeeuw et al.^11^ In our first case to discover this similarity, during neck dissection, intraoperative frozen section was performed to identify the illuminated tissue. The similarity of NIRAF intensity and pattern between normal PGs and brown adipose tissue can be misleading during PG localization in the first patients using overlay imaging via EleVision IR. However, brown adipose tissue, which is especially present in level VI, macroscopically clearly differs from PGs. In those cases, the surgeon should not get distracted by similar NIRAF patterns of brown adipose tissue and PGs.

Among our patients, parathyroid adenoma presented a heterogeneous autofluorescence pattern with more autofluorescence intensity in nonadenomatously altered parts than in adenomatously altered tissue. Demarchi et al. described the same pattern for parathyroid adenoma using the Fluobeam LX^®^ device (Fluoptics, Grenoble, France). Further, well defined autofluorescence intensity in the “cap” region of the PG, which corresponded to normal histology, was present.^28^

In patients with rHPT, similar intensity of NIRAF was observed in our study as seen in patients with pHPT, yet with a more homogenous pattern, which has also been described in previous studies.^12,29^ Compared with rHPT, PG NIRAF intensity was stronger in multigland hyperplasia due to pHPT, but still lower than in healthy PGs. McWade et al. addressed this variation by arguing that either the molecular composition or a different constitution of cell hyperplasia in rHPT lead to downregulation of fluorescence signals.^12^

In our patient collective, we also observed spots of NIRAF signals on the thyroid gland. In one (0.5%) patient, the autofluorescent spot on the thyroid gland was a hemorrhaged thyroid cyst, and in another (0.5%), the fluorescent part of the thyroid gland was a cyst with detritus in final histology. These NIRAF signals showed a similar intensity as healthy PGs but with a more diffuse autofluorescence pattern. We observed stronger NIRAF compounds in thyroid tissue in patients suffering from thyroiditis than in other diseases of the thyroid glands. Similar findings with bright spots in the thyroid gland were present in another study and were described as colloid nodules in final histology.^11^

In one (0.5%) patient with MTC, we found a lymph node metastasis on the recurrent laryngeal nerve, which showed a NIRAF intensity and pattern similar to that of normal PGs. In another study using the Fluobeam800^®^ system, and in contrast to our observations, autofluorescence was not seen in any obvious surrounding tissue of thyroid cancer or metastatic lymph nodes.^11^ In another study, NIRAF was described in lymph node metastasis in papillary carcinoma.^10^ Furthermore, after PG extirpation, the ex vivo NIRAF intensity and pattern were the same as before parathyroidectomy. This finding has been described in previous studies, confirming that depiction of NIRAF does not depend on PG vascularization.^11^

## Conclusions

In this study, we report our initial experiences with the application of NIRAF-based overlay imaging using EleVision IR and set standards for its ideal usage. Tissue showing NIRAF was localized with an ideal distance of 8 to 12 cm and an angle of 90°, considering different overlay intensity and patterns. Automatic adjustment of the NIRAF signal was performed with a mean IR% of 34.5. Currently, NIRAF-based overlay imaging has shown to be a good and clinically easily applied adjunct tool for the localization of PGs. Further prospective studies are necessary to extend and specify the standards for subgroups of patients.
